# TRIP6 promotes inflammatory damage via the activation of TRAF6 signaling in a murine model of DSS-induced colitis

**DOI:** 10.1186/s12950-021-00298-0

**Published:** 2022-01-04

**Authors:** Yun Yang, Xiu-Ming Li, Jing-Ru Wang, Yan Li, Wen-Long Ye, Yi Wang, Yu-Xuan Liu, Zhi-Yong Deng, Wen-Juan Gan, Hua Wu

**Affiliations:** 1 Department of Pathology, Medical College of Soochow University, Soochow University, Suzhou, 215123 China; 2 Department of Pathology, The First Affiliated Hospital of Soochow University, Suzhou, 215006 China; 3 Department of Pathology, The First People’s Hospital of Kunshan, Kunshan, Suzhou, 215300 China; 4 Department of Pathology, Dushu Lake Hospital Affiliated of Soochow University, Suzhou, 215124 China

**Keywords:** Inflammation, Colitis, TRIP6, TRAF6 signaling, Animal model

## Abstract

**Background:**

TRIP6 is a zyxin family member that serves as an adaptor protein to regulate diverse biological processes. In prior reports, TRIP6 was shown to play a role in regulating inflammation. However, its in vivo roles and mechanistic importance in colitis remain largely elusive. Herein, we therefore employed TRIP6-deficient (TRIP6^−/−^) mice in order to explore the mechanistic importance of TRIP6 in a dextran sodium sulfate (DSS)-induced model of murine colitis.

**Findings:**

Wild-type (TRIP6^+/+^) mice developed more severe colitis following DSS-mediated disease induction relative to TRIP6^−/−^ mice, as evidenced by more severe colonic inflammation and associated crypt damage. TRIP6 expression in wild-type mice was significantly elevated following DSS treatment. Mechanistically, TRIP6 binds to TRAF6 and enhances oligomerization and autoubiquitination of TRAF6. This leads to the activation of NF-κB signaling and the expression of pro-inflammatory cytokines such as TNFα and IL-6, in the in vivo mouse model of colitis.

**Conclusions:**

These in vivo data demonstrate that TRIP6 serves as a positive regulator of DSS-induced colitis through interactions with TRAF6 resulting in the activation of inflammatory TRAF6 signaling, highlighting its therapeutic promise as a protein that theoretically can be targeted to prevent or treat colitis.

**Supplementary Information:**

The online version contains supplementary material available at 10.1186/s12950-021-00298-0.

## Background

Inflammatory bowel disease (IBD) is a term used to refer to chronic inflammatory diseases affecting the colon and intestines, including both Crohn’s disease (CD) and ulcerative colitis (UC) [1]. The etiological basis for IBD is complex and influenced by a range of host genetic factors, immunological dysfunction, impaired function of the intestinal epithelial barrier dysfunction, and environmental perturbations [2, 3]. While a number of anti-inflammatory drugs have been developed to facilitate IBD treatment [4], the majority of affected patients fail to achieve long-lasting relief. Genome-wide association studies related analyses have led to the identification of many genes associated with IBD susceptibility loci, including NOD2, IL23R, and ATG16L1 [5–7]. We have also recently demonstrated that the orphan nuclear receptor superfamily protein Nur77 is downregulated in colon tissue samples from UC and CD patients, with Nur77-knockout mice being more susceptible to colitis [8]. These recent research advances highlight promising novel candidate biomarkers with the potential to guide future IBD patient diagnosis and treatment. Despite these efforts, however, more work is needed to identify novel potential targets for IBD treatment.

Thyroid receptor interactor protein 6 (TRIP6, also known as ZRP-1 or OIP1), is a member of the zyxin LIM protein family that contains an N-terminal proline-rich region as well as three C-terminal cysteine-rich zinc finger domains [9]. TRIP6 functions as an adaptor protein and thereby controls processes including transcription, motility, and cytoskeletal remodeling by interacting with a variety of proteins [10–13]. For example, by interacting with lysophosphatidic acid 2 (LPA2) receptor in the cytoplasm, TRIP6 can enhance lysophosphatidic acid-induced cellular migratory activity [14]. TRIP6 interactions with supervillin (SV) in the cytoplasm can also facilitate focal adhesion formation [15]. In the nucleus, TRIP6 can interact with glucocorticoid receptor (GR) to control gene transcription [13]. Recent evidence also highlights the importance of TRIP6 as a regulator of inflammatory signaling, in part owing to its ability to interact via its LIM domains with RIP2 and to thereby drive the activation of NF-κB in a manner that can be influenced by TNF and IL-1 [16]. TRIP6 can also inhibit the ability of A20 to bind to TRAF6, in turn enhancing TRAF6-mediated NF-κB activation [17]. In the glomeruli of streptozotocin-challenged mice, the expression of TRIP6 is significantly increased, and its overexpression can promote fibrosis and inflammation in podocytes exposed to high-glucose conditions [18]. Together, these prior results thus suggest that TRIP6 is an important regulator of inflammation and associated diseases processes. Whether and how TRIP6 regulates colitis, however, remains unknown.

As such, we herein analyzed the phenotypic characteristics of TRIP6^−/−^ and wild-type (WT; TRIP6^+/+^) mice in the context of a DSS-induced colitis model system. Through a series of experiments, we found that TRIP6^−/−^ mice exhibited a less serious disease phenotype relative to TRIP6^+/+^ mice, consistent with a role for TRIP6 as a regulator of colitis.

## Materials and methods

### Antibodies and reagents

Anti-IκBα was from Cell Signaling Technology (MA, USA). Anti-β-actin were purchased from Sigma-Aldrich (Sigma-Aldrich, MO, USA). Anti-TRIP6 was purchased from Santa Cruz Biotechnology (Santa Cruz, CA, USA). Anti-CD45, anti-CD11b, anti-CD4, anti-F4/80, and anti-B220 were from BD Bioscience (CA, USA). DSS was purchased from MP Biomedicals (USA). LIVE/DEAD™ Fixable Violet Dead Cell Stain Kit was from Invitrogen (USA).

### Plasmid constructs

Expression construct for Flag-TRAF6 was kindly provided by Dr. Ashley Mansell (Monash Institute of Medical Research, Australia). TRAF6 and TRIP6 were generated by PCR and inserted into the pEGFP-C1 and pCMV-Myc vectors, respectively. Cells were transiently transfected with these plasmids using Lipofectamine 2000 (Invitrogen).

### Mice

TRIP6^+/+^ and TRIP6^−/−^ mice were obtained from Cyagen (Taicang, Jiangsu, China) and were housed under specific pathogen-free conditions with a 12 h light/dark cycle and free access to standard food and water at the Laboratory Animal Center in Soochow University (China). The Animal Care and Use Committee of Soochow University approved all animal studies.

### Tissue collection and assessment

Tissue samples collected from TRIP6^+/+^ and TRIP6^−/−^ mice for analysis included the colon, mesenteric lymph nodes, spleen, and peripheral lymph nodes. Spleen weight, colon length, and the numbers of cells in the mesenteric and peripheral lymph nodes were quantified.

### DSS-induced colitis modelling

Mice (8-10 weeks old) were administered 2.5% DSS in their drinking water for 7 days, followed by a 6-day recovery period during which they were administered normal water. All mice were weighed daily, and were euthanized at experimentally appropriate time points. Colon tissue samples were then collected, and colitis severity was assessed by staining paraffin-embedded colon sections with hematoxylin and eosin (H&E), with inflammation and crypt damage being quantified and given a score ranging from 0 to 4 as indicated in Supplemental Table 1.

### Histological staining

H&E staining was performed as in previous reports [19].

### qPCR

Trizol LS (Invitrogen) was used to extract total RNA from appropriate samples, after which a RevertAid™ First Strand cDNA Synthesis Kit (Fermentas) was used to synthesize cDNA. qPCR was then performed with a SYBR Green Power Master Mix based upon provided instructions (Applied Biosystems), using the primers listed in Supplemental Table 2.

### Western blotting

Samples of full-thickness colon tissue were homogenized in a 1 mL volume of cell lysis buffer supplemented with a protease inhibitor cocktail (Roche, Mannhein, Germany) for 30 min on ice, after which homogenates were centrifuged for 20 min at 13,000 xg at 4 °C. Equal amounts of protein were then separated via 10% SDS-PAGE and transferred to PVDF membranes, after which protein expression was detected using appropriate antibodies, with signaling being visualized with an ECL system.

### Immunoprecipitation and ubiquitination assay

Cells were lysed in a buffer containing Tris-HCl (20 mM, pH 7.4), EDTA (10 mM), NaCl (100 mM), and IGEPAL (1%). Whole-cell lysates were subjected to immunoprecipitation with the indicated antibodies using protein A/G beads (GE Healthcare). For the detection of TRAF6 ubiquitination, 10 mM N-ethylmaleimide was included in the lysis buffer.

### Luciferase reporter assay

An NF-κB reporter plasmid with an NF-κB response element was transfected into RAW264.7 cells using Lipofectamine 2000 (Invitrogen). After 24 h, the cells were treated with LPS (10 ng/ml) for 12 h. Cells were lysed and reporter activity was analyzed with the Luciferase Reporter Assay system (Promega). Each transfection also included β-gal as a normalization control.

### ELISAs

TNFα and IL-6 levels were assessed using mouse-specific ELISA kits (R&D Systems) based on provided directions.

### Flow cytometry

Colon tissue samples were isolated, rinsed three times using cold PBS, and digested for 45 min at 37 °C in DMEM containing 2% FBS, collagenase IV (1 mg/mL; Sigma-Aldrich), and DNase I (10 U/mL; Roche). The resultant digest was then passed through a 70-μm mesh filter to generate a single-cell suspension, which was stained with LIVE/DEAD, anti-CD45, anti-CD11b, anti-F4/80, anti-CD4, anti-CD8, and anti-B220 prior to analysis using a BD LSR Fortessa™ Flow Cytometer. All data were analyzed using NovoExpress or FlowJo X (TreeStar, CA, USA).

### Statistical analysis

Data are given as means±SD. Statistical significance was analyzed using Student’s t-tests (unpaired, two-tailed). Experiments were repeated in triplicate and analyzed using Prism 5.03 software (GraphPad Software), with *p* < 0.05 as the significance threshold (*p < 0.05; **p < 0.01; ***p < 0.001; *ns*, not significant).

## Results and discussion

Recent research evidence suggests that TRIP6 can control inflammatory signaling [16, 18], yet its in vivo function in inflammatory contexts has yet to be evaluated. Herein, we thus examined the functional role of TRIP6 in vivo by evaluating inflammatory and immune cells within the immunological organs of TRIP6^+/+^ and TRIP6^−/−^ mice at 3 months of age. Relative to TRIP6^+/+^ mice, TRIP6^−/−^ animals did not exhibit any changes in cell numbers in their mesenteric lymph nodes (Fig. S1a), peripheral lymph nodes (Fig. S1b), or spleens (Fig. S1c). There were also no observed differences in colon length when comparing TRIP6^+/+^ and TRIP6^−/−^ mice (Fig. S1d). This suggests that TRIP6 deficiency on its own has no adverse impact on normal physiological function in these mice. To establish whether the loss of TRIP6 would alter inflammatory or immune cell activity in further detail, we performed a flow cytometry-mediated analysis of these cell populations within colon tissue samples from TRIP6^+/+^ and TRIP6^−/−^ mice. Our analysis revealed no significant differences in the frequencies of colon-infiltrating macrophages (F4/80^+^ and CD11b^+^), T cells (CD4^+^ and CD45^+^), or B cells (CD45^+^ and B220^+^) when comparing these groups (Fig. S1e-g). TRIP6 deficiency also had no impact on colon macrophage-mediated TNFα secretion (Fig. S1h). Together, these results suggested that TRIP6 deficiency had no adverse impact on inflammation or immune function under normal physiological conditions.

**Fig. 1:**
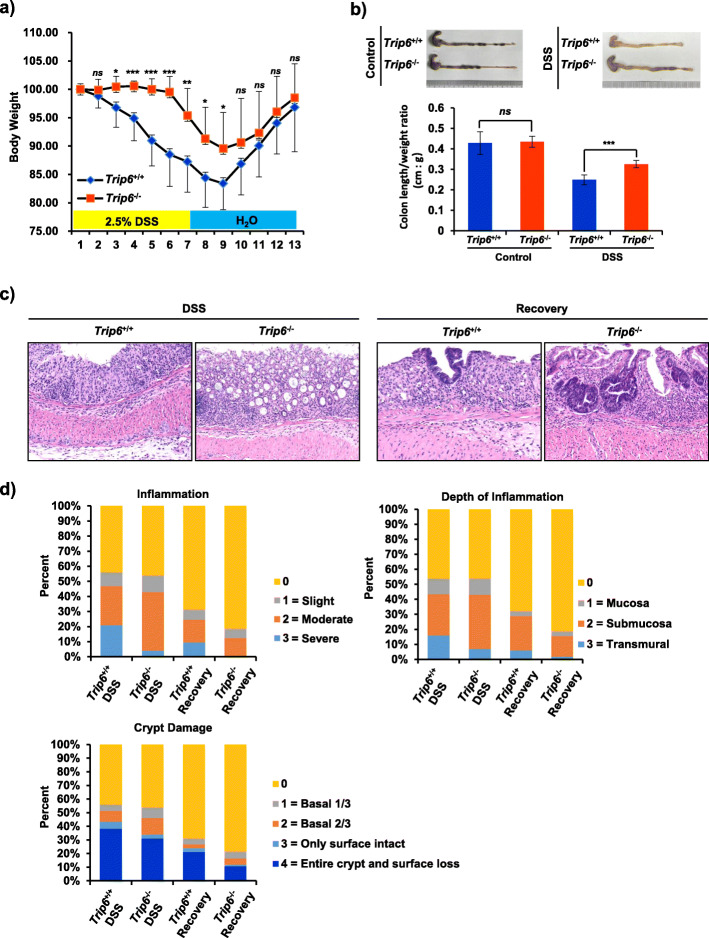
Knocking out TRIP6 enhances murine resistance to DSS-induced colitis. **a** Weight loss was monitored in TRIP6^+/+^ and TRIP6^−/−^ mice relative to baseline following treatment with 2.5% DSS and during a subsequent 6-day recovery period in which mice were allowed to drink regular water (*n* = 12/group). Data are means ± SD. *p < 0.05; **p < 0.01; ***p < 0.001, *ns*, not significant. **b** Representative images of colon tissues are shown (top) and colon length is represented as a ratio (cm:g) relative to the starting weight of mice prior to DSS administration (bottom). Data are means ± SD. *ns*, not significant; ***p < 0.001. **c** H&E staining was used to assess TRIP6^+/+^ and TRIP6^−/−^ murine colon tissue sections on days 7 and 13 following DSS treatment, with representative images being shown. **d** Inflammation, depth of inflammation, and crypt damage were scored for samples of colon tissue from TRIP6^+/+^ and TRIP6^−/−^ mice following treatment with DSS

Next, we assessed the role of TRIP6 in the context of inflammatory pathology by establishing a DSS-induced model of murine colitis. To that end, TRIP6^+/+^ and TRIP6^−/−^ mice were administered DSS-containing drinking water (2.5% w/v) for 7 days, after which a recovery period during which they were administered regular drinking water for 6 days was conducted, with body weight being recorded daily. By day 3 following DSS treatment, TRIP6^+/+^ mice began to lose more weight than did TRIP6-deficient mice, and this continued until day 7 of DSS administration. During the following recovery period, we still observed significant body weight loss in the TRIP6^+/+^ mice compared to the TRIP6^−/−^ mice until day 9, while both cohorts of animals exhibited similar weights from day 10 onwards (Fig. 1a). These results suggested that TRIP6^+/+^ mice are susceptible to DSS-colitis and experience impaired recovery. Consistent with these results, DSS-treated TRIP6^+/+^ mice exhibited a significant reduction in the colon length/weight ratio (*p* = 0.0004) as compared to DSS-treated TRIP6^−/−^ mice (Fig. 1b). Histopathological analyses of inflammation, depth of inflammation, and crypt damage further revealed significantly higher pathological scores as indicated by the fact that TRIP6^+/+^ mice exhibited more severe inflammation and crypt damage, as well as a greater depth of inflammation as compared to TRIP6^−/−^ mice on days 7 and 13 of the study period (Fig. 1c-d). In line with these findings, H&E staining showed that CD68-positive cells (inflammatory cells, such as monocytes and macrophages) were increased in DSS-treated TRIP6^+/+^ mice as compared to TRIP6^−/−^ mice (Fig. S2a and b), suggesting that more severe colitis, epithelial injury, and the overactivation of inflammatory cells were evident in DSS-treated TRIP6^+/+^ mice as compared to TRIP6^−/−^ mice. These findings indicate that TRIP6 can serve as a positive regulator capable of promoting colitis in a DSS-induced model of murine colitis.

**Fig. 2:**
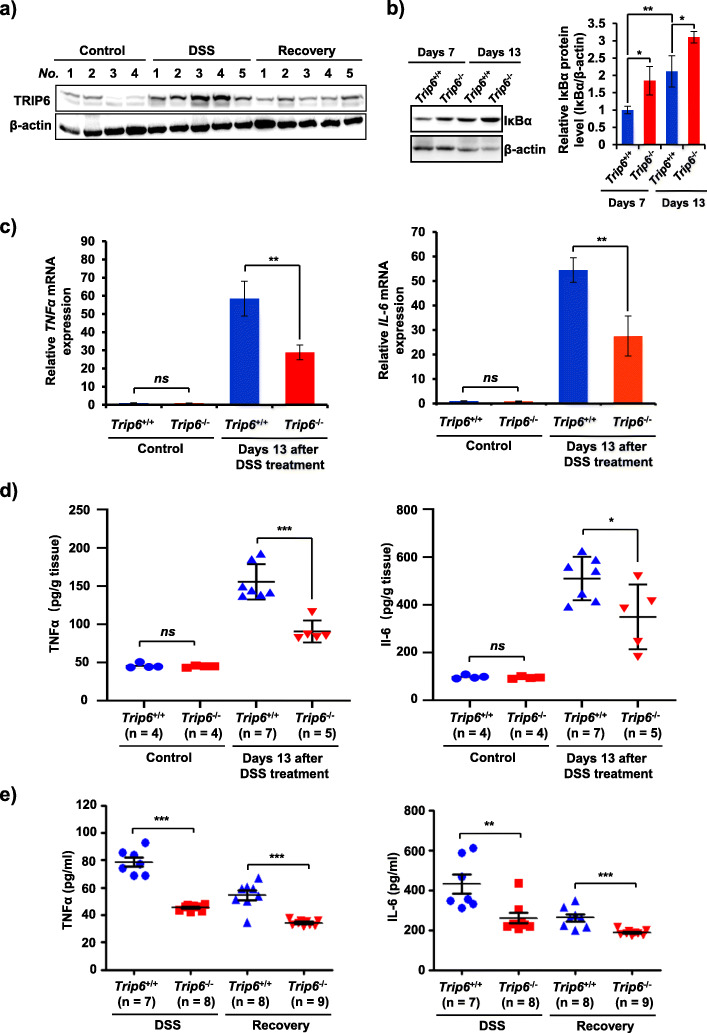
TRIP6 deficiency impairs inflammatory NF-κB signaling activity in a DSS-induced model of experimental murine colitis. **a** Western blotting was used to assess the expression of TRIP6 in colon tissues from TRIP6^+/+^ mice following DSS treatment, with representative images being shown. **b** Western blotting was used to assess the expression of IκBα in colon tissues from TRIP6^+/+^ and TRIP6^−/−^ mice following DSS treatment, with representative images and being shown (left). Protein levels of IκBα were quantified by using densitometry and normalized to those of β-actin for comparison (Right). Data are means ± SD. *n* = 3, *p < 0.05 and **p < 0.01. **c**-**d** qPCR (**c**) and ELISAs (**d**) were respectively used to evaluate TNFα and IL-6 mRNA and protein levels in colon tissues. **e** ELISAs were used to evaluate serum TNFα and IL-6 levels in TRIP6^+/+^ and TRIP6^−/−^ mice over the course of DSS induction and recovery. Data are means ± SD. **p < 0.01; **p < 0.01; ***p < 0.001; *ns*, not significant

Given the observed role of TRIP6 in regulating inflammation in vivo, we next sought to investigate the underlying mechanism by which TRIP6 promotes colitis in the DSS-induced mouse model system. Interestingly, we found that TRIP6 expression was regulated by DSS exposure. Indeed, TRIP6 protein levels rose markedly in the colon tissues of TRIP6^+/+^ mice over the course of DSS administration (Fig. 2a), further supporting a role for this adaptor protein in colitis. These results are in line with prior evidence suggesting that ectopic TRIP6 expression can reverse the impact of PTPN14 silencing-mediated inhibition of fibrosis and inflammatory responses [18]. However, how the expression of TRIP6 is regulated in the context of colitic disease pathogenesis remains to be investigated. Given the evidence that nuclear transcription factor kappa B (NF-κB) is one of the key regulators of the pathogenesis of IBD, and inhibition of NF-κB activation has been explored as an important therapeutic strategies for IBD [20], we thus sought to test whether TRIP6 was implicated in the regulation of NF-κB signaling in colitis. We first determined the effect of TRIP6 on expression of IκBα, a key inhibitor of NF-κB activation [21]. Western blotting assays revealed that the loss of TRIP6 expression in mice was associated with the pronounced inhibition of IκBα degradation over the course of DSS treatment and recovery (Fig. 2b), indicating a role for TRIP6 as an enhancer of in vivo NF-κB signaling in this model of experimental colitis. These results are consistent with prior findings demonstrating that TRIP6 can activate inflammatory NF-κB signaling, thereby promoting resistance to apoptotic death and cellular invasion in vitro [16]. Inflammatory cytokine and chemokine production is a hallmark of colitis [22]. We thus further assessed TNFα and IL-6 expression in these experimental animals, revealing that TRIP6^−/−^ mice exhibited decreased colon tissue TNFα and IL-6 mRNA (Fig. 2c) and protein (Fig. 2d) levels relative to TRIP6^+/+^ mice. We similarly observed significant reductions in serum TNFα and IL-6 levels in TRIP6^−/−^ mice relative to TRIP6^+/+^ mice during the DSS treatment and recovery periods (Fig. 2e). These data thus demonstrate that TRIP6^+/+^ mice are highly susceptible to DSS-induced colitis, whereas the loss of TRIP6 expression compromises inflammatory signaling activity in this pathological context.

**Fig. 3:**
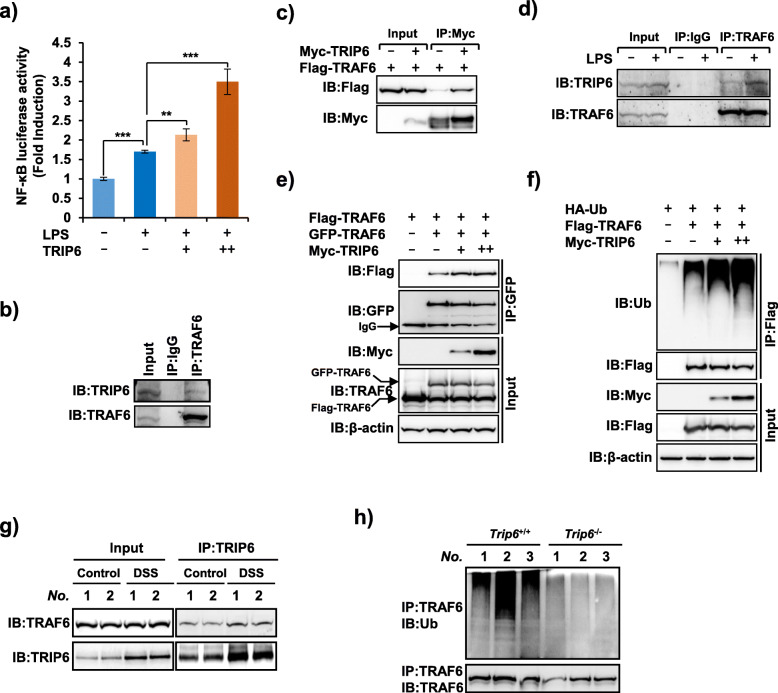
TRIP6 activates NF-κB signaling through interaction with TRAF6 and regulation of TRAF6 oligomerization and autoubiquitination. **a** A luciferase reporter assay was used to measure NF-κB reporter activity in TRIP6-transfected RAW264.7 cells treated with LPS (10 ng/ml). **b**, **c** TRIP6 interacts with TRAF6. Immunoprecipitation (IP) and immunoblot (IB) analyses of cell lysates of THP-1 cells (**b**) and HEK293T cells transfected with Myc–TRIP6 and Flag–TRAF6 (**c**). **d** LPS enhances the interaction between TRIP6 and TRAF6. IP and IB analyses of cell lysates of THP-1 cells treated with LPS (50 ng/ml). **e**, **f** TRIP6 promotes the oligomerization and autoubiquitination of TRAF6. HEK293T cells were transfected with the indicated plasmids for 30 h, the lysates were analyzed by IP with anti-GFP antibody (**e**) or anti-Flag antibody (**f**), and the oligomerization and autoubiquitination of TRAF6 were examined via Western blotting. **g** Interactions between TRIP6 and TRAF6 were analyzed by IP in intestinal samples from wild-type mice treated with or without DSS. **h** IP of endogenous TRAF6 from colon lysates of DSS-treated TRIP6^+/+^ and TRIP6^−/−^ mice was performed, followed by the IB analysis of TRAF6 autoubiquitination using an anti-ubiquitin antibody

Tumor necrosis factor receptor-associated factor 6 (TRAF6) is an intracellular signaling molecule that plays crucial functions in controlling the activation of the canonical NF-κB pathway initiated by TLR and IL-1R family proteins [23]. Upon activation of the TLR-IL-1R signaling, TRAF6 is recruited to appropriate receptor complexes wherein it mediates the activation of downstream signaling proteins including NF-κB and AP- 1[24]. Here, we found that overexpression of TRIP6 substantially increased lipopolysaccharide (LPS)-induced NF-κB activity in a dose-dependent manner (Fig. 3a). Recent studies have shown that TRIP6 recruits TRAF6 to the LPA2 receptor and promotes lysophosphatidic acid-induced NF-κB activation [17], however, whether TRIP6 participates in the pathogenesis of colitis through interaction with TRAF6 remains unknown. Our results revealed that TRIP6 contributes to the activation of NF-κB signaling through its interaction with TRAF6. As shown in Fig. 3b, immunoprecipitation of TRAF6 in THP-1 cells using an anti-TRAF6 antibody resulted in the coimmunoprecipitation of a significant amount of TRIP6 protein. Consistently, the interaction between TRIP6 and TRAF6 was detectable in the exogenous system (Fig. 3c). Taken together, these results show that TRIP6 can interact with TRAF6. Our findings further showed that the interaction between TRIP6 and TRAF6 can be regulated by TLR signaling. As shown in Fig. 3d, LPS greatly enhanced the endogenous interaction between TRIP6 and TRAF6. The oligomerization and autoubiquitination of TRAF6 are essential for NF-κB signal transduction [8, 25, 26]. Although TRIP6 does not exhibit deubiquitinase activity, our results revealed that TRIP6 could enhance TRAF6 oligomerization (Fig. 3e) and autoubiquitination (Fig. 3f) through its interaction with TRAF6. Consistently, in the DSS-induced model of murine colitis, the interaction between TRIP6 and TRAF6 was enhanced during DSS treatment periods (Fig. 3g). However, TRIP6 deficiency markedly impaired the ubiquitination of endogenous TRAF6 in colon tissues prepared from TRIP6^−/−^, but not wild-type mice (Fig. 3h), indicating that TRIP6 is a critical regulator in the murine model of DSS-induced colitis through its ability to regulate TRAF6 signaling.

Together, our data not only suggest that TRIP6 deficiency does not result in altered inflammatory activity or immune functionality under normal physiological conditions, but also offer robust experimental evidence for the role of TRIP6 as a positive regulator in the pathogenesis of colitis wherein it functions by promoting the infiltration of inflammatory cells in the colon tissue and the activation of TRAF6-mediated inflammatory NF-κB signaling. In summary, our results highlight a previously unknown role for TRIP6 as a regulator of intestinal homeostasis and underscore its promise as a therapeutic target in colitis patients.

## Supplementary Information

**Additional file 1: Figure S1.** TRIP6-knockout mice do not exhibit altered inflammatory activity or immune functionality under normal physiological conditions. **(a-c)** Gross appearance (left) and cell numbers (right) for mesenteric lymph nodes (a), peripheral lymph nodes (b), and spleens (c) from 3-month-old TRIP6^+/+^ and TRIP6 ^−/−^ mice (*n* = 4/group). **(d)** Representative colon images (left) and colon length measurements (right) for 3-month-old TRIP6^+/+^ and TRIP6^−/−^ mice (*n* = 4/group). **(e-g)** Flow cytometry was used to analyze colon-infiltrating macrophages (e), T cells (f), and B cells (g) in 3-month-old TRIP6^+/+^ and TRIP6^−/−^ mice (*n* = 6/group), with representative plots being shown. **(h)** TNFα expression in colon-infiltrating macrophages from 3-month-old TRIP6^+/+^ and TRIP6^−/−^ mice (*n* = 6/group) was assessed via flow cytometry, with representative plots being shown. **Figure S2.** TRIP6^+/+^ mice exhibit increased macrophage influx in the context of DSS-induced colitis. **(a-b)** Immunostaining for the infiltration of macrophages and monocytes using a CD68-specific antibody in the colon tissues of DSS-treated TRIP6^+/+^ and TRIP6^−/−^ mice. Representative images are shown (a), and the relative CD68-positive cell numbers were calculated (b). Data are means ± SD. *p < 0.05.

**Additional file 2: Table S1.** Primers for qPCR.

**Additional file 3: Table S2.** Histological grading of colitis.

## Data Availability

The datasets used and/or analyzed during the current study are available from the corresponding author on reasonable request.

## Abbreviations

TRIP6: Thyroid receptor interactor protein 6
TRAF6: Tumor necrosis factor receptor associated factor 6
LPS: Lipopolysaccharide
ELISA: Enzyme Linked Immunosorbent Assay
TNFα: Tumor necrosis factor alpha
IL-6: Interleukin-6
