# Supplementary figures and images for: TRIP6 promotes inflammatory damage via the activation of TRAF6 signaling in a murine model of DSS-induced colitis
Source: J Inflamm (Lond). 2022 Jan 4;19:1. doi: 10.1186/s12950-021-00298-0 (PMC8725398; doi:10.1186/s12950-021-00298-0)

# Figure S1

a)

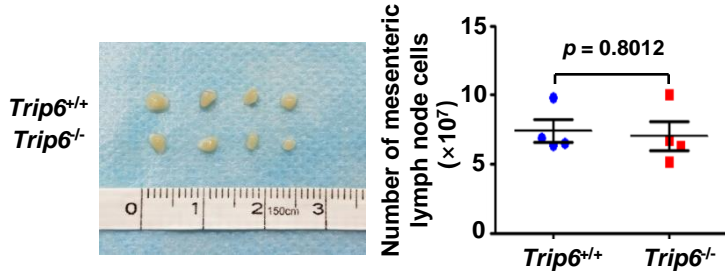

b)

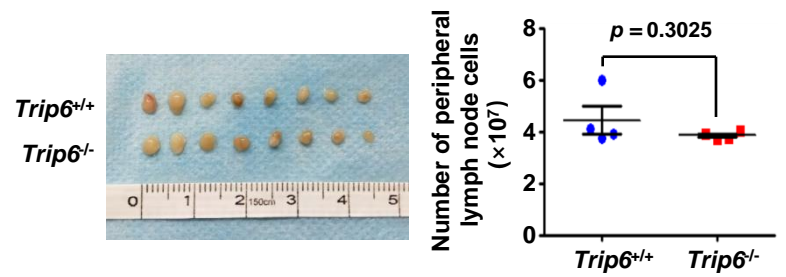

c)

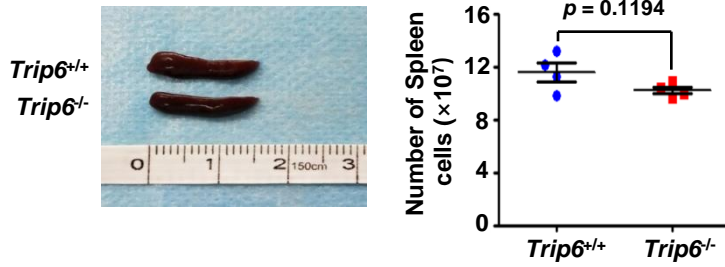

d)

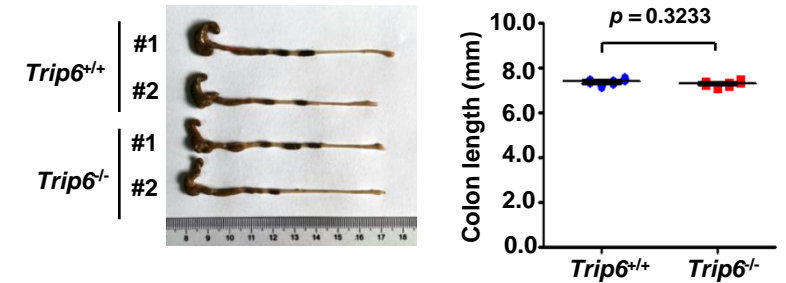

e)

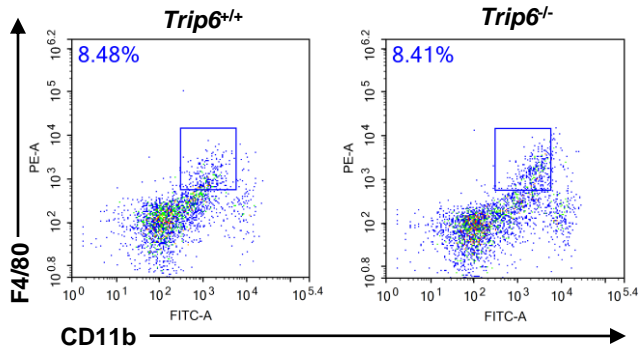

f)

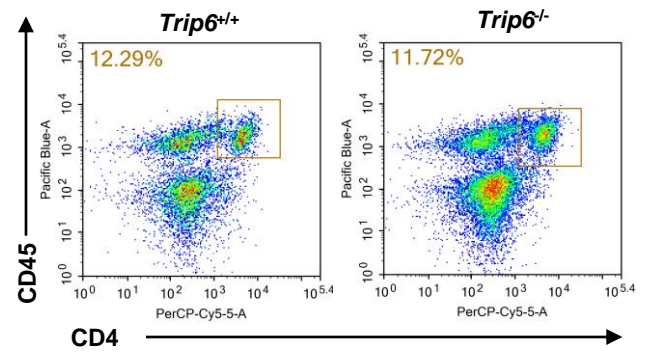

g)

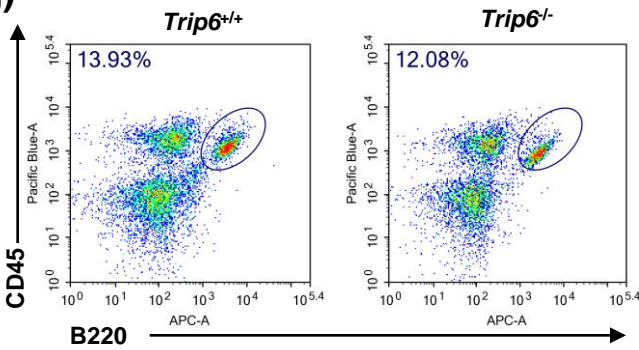

h)

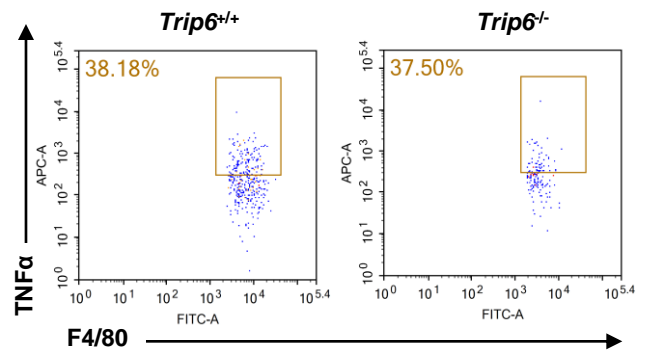

# Figure S2

a)

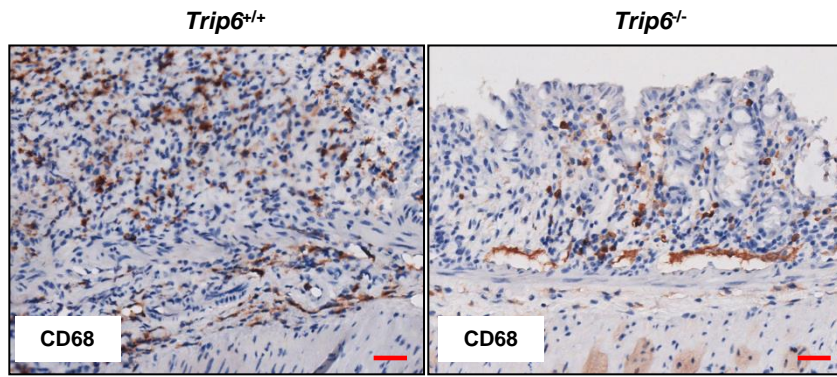

b)

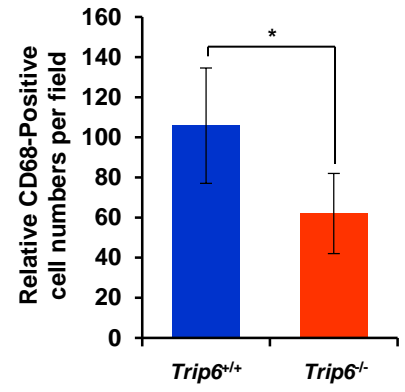

Supplement: Supplementary file 1 — Additional file 1: Figure S1. TRIP6-knockout mice do not exhibit altered inflammatory activity or immune functionality under normal physiological conditions. (a-c) Gross appearance (left) and cell numbers (right) for mesenteric lymph nodes (a), peripheral lymph nodes (b), and spleens (c) from 3-month-old TRIP6+/+ and TRIP6 −/− mice (n = 4/group). (d) Representative colon images (left) and colon length measurements (right) for 3-month-old TRIP6+/+ and TRIP6−/− mice (n = 4/group). (e-g) Flow cytometry was used to analyze colon-infiltrating macrophages (e), T cells (f), and B cells (g) in 3-month-old TRIP6+/+ and TRIP6−/− mice (n = 6/group), with representative plots being shown. (h) TNFα expression in colon-infiltrating macrophages from 3-month-old TRIP6+/+ and TRIP6−/− mice (n = 6/group) was assessed via flow cytometry, with representative plots being shown. Figure S2. TRIP6+/+ mice exhibit increased macrophage influx in the context of DSS-induced colitis. (a-b) Immunostaining for the infiltration of macrophages and monocytes using a CD68-specific antibody in the colon tissues of DSS-treated TRIP6+/+ and TRIP6−/− mice. Representative images are shown (a), and the relative CD68-positive cell numbers were calculated (b). Data are means ± SD. *p < 0.05. [file 12950_2021_298_MOESM1_ESM.pdf]
