# Supplementary material for: TRIP6 promotes inflammatory damage via the activation of TRAF6 signaling in a murine model of DSS-induced colitis
Source: J Inflamm (Lond). 2022 Jan 4;19:1. doi: 10.1186/s12950-021-00298-0 (PMC8725398; doi:10.1186/s12950-021-00298-0)
Supplement: Supplementary file 2 — Additional file 2: Table S1. Primers for qPCR. [file 12950_2021_298_MOESM2_ESM.pdf]

Supplementary Table 1: Primers for qPCR

Mouse Gene Name	Forward/Reverse
**TNFα**	F: 5'-CTCACACTCAGATCATCTTCTC-3'<br>R: 5'-CTTTCTCCTGGTATGAGATAGC-3'
**IL-6**	F: 5'-TTCCATCCAGTTGCCTTCTTG-3'<br>R: 5'-AGGTCTGTTGGGAGTGGTATC-3'
**β-actin**	F: 5'-TGGAATCCTGTGGCATCCATGAAAC-3'<br>R: 5'-TAAAACGCAGCTCAGTAACAGTCCG-3'
