# Supplementary material for: TRIP6 promotes inflammatory damage via the activation of TRAF6 signaling in a murine model of DSS-induced colitis
Source: J Inflamm (Lond). 2022 Jan 4;19:1. doi: 10.1186/s12950-021-00298-0 (PMC8725398; doi:10.1186/s12950-021-00298-0)
Supplement: Supplementary file 3 — Additional file 3: Table S2. Histological grading of colitis. [file 12950_2021_298_MOESM3_ESM.pdf]

Supplementary Table 2: Histological grading of colitis

Feature graded	Grade	Description
Inflammation	0	None
	1	Slight
	2	Moderate
	3	Severe
Depth of Inflammation	0	None
	1	Mucosa
	2	Submucosa
	3	Transmural
Crypt Damage	0	None
	1	Basal 1/3 damaged
	2	Basal 2/3 damaged
	3	Only surface epithelium intact
	4	Entire crypt and epithelium lost
